# When stem cells meet COVID-19: recent advances, challenges and future perspectives

**DOI:** 10.1186/s13287-021-02683-1

**Published:** 2022-01-10

**Authors:** Shasha Li, Hecheng Zhu, Ming Zhao, Weidong Liu, Lei Wang, Bin Zhu, Wen Xie, Cong Zhao, Yao Zhou, Caiping Ren, Hui Liu, Xingjun Jiang

**Affiliations:** 1 Cancer Research Institute, Department of Neurosurgery, National Clinical Research Center for Geriatric Disorders, Xiangya Hospital, Central South University, Changsha, 410008 China; 2 Changsha Kexin Cancer Hospital, Changsha, 410205 China; 3 The Key Laboratory of Carcinogenesis of the Chinese Ministry of Health and the Key Laboratory of Carcinogenesis and Cancer Invasion of the Chinese Ministry of Education, School of Basic Medicine, Central South University, Changsha, 410008 China; 4 School of Ophthalmology and Optometry and Eye Hospital, Wenzhou Medical University, Wenzhou, 325027 China; 5 Department of Neurosurgery, Xiangya Hospital, Central South University, Changsha, 410008 China

**Keywords:** COVID-19, SARS-CoV-2, Stem cell threapy, MSCs, Organoid models

## Abstract

Coronavirus disease 2019 (COVID-19) caused by the novel severe acute respiratory coronavirus 2 is currently spreading throughout the world with a high rate of infection and mortality and poses a huge threat to global public health. COVID-19 primarily manifests as hypoxic respiratory failure and acute respiratory distress syndrome, which can lead to multiple organ failure. Despite advances in the supportive care approaches, there is still a lack of clinically effective therapies, and there is an urgent need to develop novel strategies to fight this disease. Currently, stem cell therapy and stem cell-derived organoid models have received extensive attention as a new treatment and research method for COVID-19. Here, we discuss how stem cells play a role in the battle against COVID-19 and present a systematic review and prospective of the study on stem cell treatment and organoid models of COVID-19, which provides a reference for the effective control of the COVID-19 pandemic worldwide.

## Background

Since December 2019, surveillance of influenza and related diseases has been carried out in Wuhan, China, and a number of cases of viral pneumonia have been found, all of which were diagnosed as viral pneumonia or pulmonary infection [1]. On January 12, 2020, the World Health Organization (WHO) officially named it 2019-nCoV [2]. The virus was later renamed as severe acute respiratory syndrome coronavirus (SARS-CoV-2) by the coronavirus Research Group, and the WHO named the disease caused by this virus COVID-19. Subsequently, the WHO also classified the outbreak as a public health emergency of international concern and declared a global pandemic. As of 26 November 2021, 259,502,031 confirmed cases and 5,183,003 deaths have been reported globally (www.WHO.int).

Coronaviruses are pathogenic microorganisms that pose a serious threat to human and animal health and are known to cause colds and more serious diseases, such as Middle East respiratory syndrome (MERS) and severe acute respiratory syndrome (SARS). SARS-CoV-2 is a novel coronavirus strain that has never been found in humans before [3, 4]. Since 2003, research on SARS and MERS coronaviruses has never stopped, and certain progress has been made in its natural origin and pathogenic mechanism. To date, however, there is no specific treatment for SARS-CoV, MERS-CoV, SARS-CoV-2 and other HCoV infections. Fortunately, the COVID-19 vaccine has been marketed with the joint efforts of various countries. Upon vaccination, the body can be stimulated to produce corresponding protective antibodies, thereby mitigating the risk of infection with SARS-CoV-2. Nevertheless, SARS-CoV-2 virus remains cryptic up to now as its many features including transmission, infection, and treatment are still waiting for unraveling [5]. The virus has been undergone various mutations that may influence the effectiveness of antibodies [6, 7]. Therefore, finding safe and effective treatments for COVID-19 has always been our goal.

Stem cells, especially mesenchymal stem cells (MSCs), have powerful immune regulation and tissue damage repair functions. In recent years, MSCs and lung stem/progenitor cells (LSCs) have been widely used in the treatment of viral infections and various diseases, including acute lung injury (ALI) [8]. Mesenchymal stem cell (MSC) therapy has also been in the spotlight since the COVID-19 outbreak [9]. Current studies have shown that MSCs can effectively reduce the severe inflammatory response in patients caused by SARS-CoV-2, reduce lung injury, improve lung function, protect and repair the lung, and play a positive role in alleviating pulmonary fibrosis in COVID-19 patients.

In addition, many studies have shown that a variety of organoids derived from stem cells provide an ideal and sufficient model for exploring the possibility and mechanism of SARS-CoV-2 infecting multiple organs, which can better serve clinical treatment research. Here, we discuss how stem cells will play a role in the battle against COVID-19 and present a systematic review and prospective of the study on stem cell-based therapy and disease modeling for COVID-19.

## Overview of SARS-CoV-2 infection (COVID-19)

**Fig. 1:**
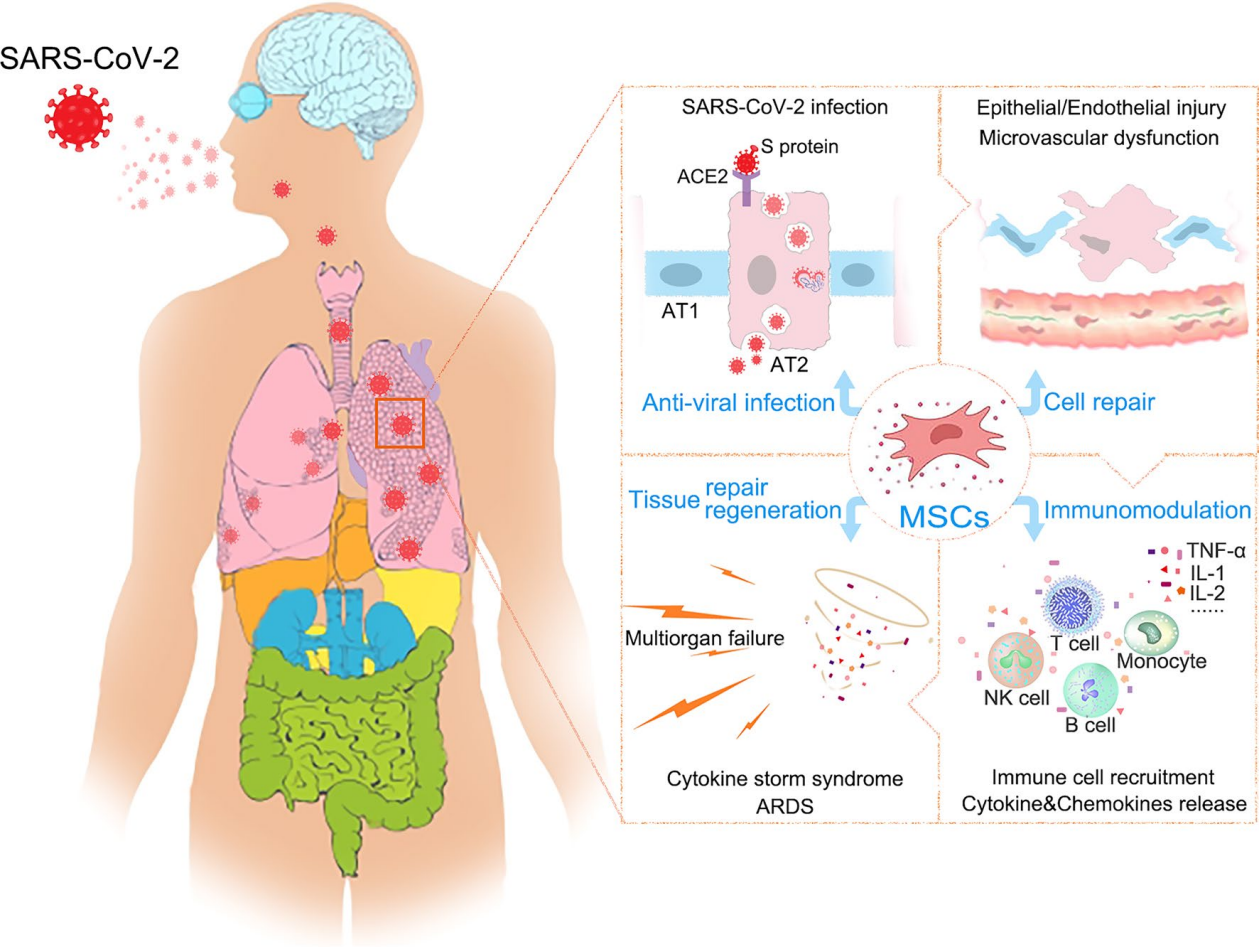
Schematic diagram illustrating COVID-19 pathophysiology and the potential mechanisms of MSC therapy

Coronaviruses are a class of enveloped plus stranded RNA viruses that can cause severe respiratory, digestive and nervous system diseases in humans and various animals [10–12]. On the basis of phylogenetic analyses of viral nucleic acid sequences, the International Committee on taxonomy of viruses divides coronavirus into four genera:α、β、γ and δ. Among them, the β coronavirus are the most highly infectious and pathogenic, and they have led to the outbreak of the severe respiratory syndrome (SARS, SARS-CoV infection) in 2003, the Middle East respiratory syndrome (MERS, MERS-CoV infection) in 2012 and the Coronavirus disease in 2019 (COVID-19, SARS-CoV-2 infection). The genome of the SARS-CoV-2 virus consists of 29,891 base pairs and encodes 9,860 amino acids. It has approximately a 79% and 50% identity with the nucleotide sequences of SARS-CoV and MERS-CoV, respectively [13, 14]. The diameter of SARS-CoV-2 virus is approximately 80–120 nm, and it can encode 29 proteins, among which the spike glycoprotein (S protein) expressed on viral envelopes is an essential structural protein to mediate the invasion of the virus into host cells [15]. After the S protein of SARS-CoV-2 binding with the angiotensin converting enzyme 2 (ACE2) receptor of host cells, the virus is absorbed and enters into the host cells and then replicates and spreads in large quantities (Fig. 1). The main target cells for SARS-CoV-2 infection are ACE2-positive cells, such as type II alveolar epithelial (AT2) cells and resident alveolar macrophages in the lung, and the endothelial cells in the liver, kidney, hearts and intestines [16–20].

Symptoms of COVID-19 vary widely among individuals, ranging from asymptomatic infection to critical illness. A medical observation of 47 patients in the early stage of the epidemic found that fever, fatigue and dry cough were the main manifestations of COVID-19, and a few patients had expectoration and diarrhea [21, 22]. Most severe cases develop dyspnea or hypoxemia within a week and even rapidly developed ARDS, septic shock, metabolic acidosis and coagulation dysfunction in severe cases [21, 23, 24]. COVID-19 symptoms occur after an incubation period of approximately 5.2 days of infection, and the time from symptom onset to death ranges from 6 to 41 days [25]. Through a retrospective analysis of the reported cases, it was found that the age of the confirmed cases was between 30 and 69 years old (77.8%), and male cases accounted for 51.4%. The death cases were mainly people aged 60 years or above, and they originally suffered from cardiovascular diseases, hypertension and other basic diseases. Studies have confirmed that human respiration is an important mode of transmission for COVID-19, but it is not the only way to spread the infection. Moreover, aerosol transmission and gastrointestinal transmission have also been reported [26, 27]. The exposure of the mucous membranes and unprotected eyes increases the risk of infection, and newborns may also be infected while in the uterus or during the perinatal period [28–31].

Although several vaccines have been successfully marketed and successively given to people, COVID-19 has not been fully controlled globally. The effectiveness and safety of vaccines are still issues that need to be concerned. And similar with other viruses, SARS-CoV-2 accumulates nucleotide mutations over time [32]. Along with its further diffusion, more variants may continue to emerge, which may be subject to selective pressures from natural immunity, vaccines and therapeutic drugs. To complement the role of vaccines, the treatment of COVID-19 still needs to be continuously explored. Among these potential treatments, stem cell therapy has received extensive attention as a new treatment method.

## Stem cells for COVID-19 Therapy

Cell therapy is a significant treatment that has been applied in a variety of diseases, including lung [33–37], cardiovascular [38–40], liver [41, 42], kidney [43, 44] and other diseases [45–48]. Stem cells are primitive cells with self-renewal ability and multidirectional differentiation potential and can differentiate into a variety of functional cells or tissues. Stem cell therapy involves cultivating new, normal, younger cells, tissues and even micro organs through isolation, culture, and directed differentiation of the stem cells in vitro and then transplanting them to specific parts of the body instead of the cells that are damaged or have died, thereby restoring body functions. Stem cell therapy is available not only for severe COVID-19 patients but also to those who have recovered from severe COVID-19 complications to repair damaged lungs, making it an ideal treatment.

Currently, there are many applications and studies for experimental stem cell therapy in critically ill patients with COVID-19 (Table 1), especially MSC therapy [49]. MSCs are derived from the mesoderm and ectoderm at the early stage of embryonic development and have attracted increasing attention due to their multidirectional differentiation potential, immunomodulatory properties and lack of ethical controversy [50–52]. With the development of regenerative medicine and precision medicine, MSCs have been isolated from different tissues, and used for specific tissue repair and regeneration [53]. To date, MSCs can be obtained from a variety of adult tissues, mainly bone marrow, umbilical cord blood, adipose tissue, endometrium, uterine blood, embryos, etc. [54–56].

**Table 1:** Stem cells for COVID-19 therapy

Stem cell sources	Study design	Population	Key findings	References
BMSCs (allogeneic)	Pilot study	7 COVID-19 Patients (1 Critically severe, 4 severe, 2 common type)	The peripheral lymphocytes were increased, the C-reactive protein decreased, and the overactivated cytokine-secreting immune cells CXCR3^+^CD4^+^ T cells, CXCR3^+^CD8^+^ T cells, and CXCR3^+^ NK cells disappeared in 3–6 days	Leng et al. [57]
hUC-MSCs (allogeneic)	Case report	65-year-old COVID-19 patient with severe pneumonia,respiratory failure and multiorgan failure	Vital signs stabilized, not dependent on ventilator. After the infusion patient was negative for the virus on throat swabs after 2 days	Alturi et al. [58]
hUC-MSCs (allogeneic)	Phase I clinical trial	18 COVID-19 moderate and severe ill	The PaO_2_/FiO_2_ ratio improved; Lung lesions of patients were well controlled within 6 days, and completely disappeared within 2 weeks	Wang et al. [59]
hUC-MSCs (allogeneic)	Case report	12 COVID-19 critically ill	Clinical symptoms, including weakness and fatigue, shortness of breath, and low oxygen saturation were improved	Shu et al. [60]
hUC-MSCs (allogeneic)	Case report	66-year old female	Absolute lymphocyte count was improved after twice administration of convalescent plasma and no infusion or allergic reactions were seen after hUC-MSC administration	Peng et al. [61]
hUC-MSCs (allogeneic)	Case report	65-year-old critically-ill woman	The T-cell ounts normalized and initial therapy of α-thymosin when combined with hUCMSCs greatly reduced the inflammation	Liang et al. [62]
human umbilical cord Wharton's jelly-derived MSCs (hWJCs) (allogeneic)	Case report	Critically ill 54-year old male patient having cough, fever and tightness of chest from 4 days	After treatment, the percentage and counts of lymphocyte subsets (CD3^+^, CD4^+^, and CD8^+^ T cell) were increased, and the level of IL-6, TNF-α, and C-reactive protein is significantly decreased after hWJC treatment	Zhang et al. [63]
AT-MSCs (allogeneic)	Case report	13 severe COVID-19 pneumonia patients	Administration of AT-MSCs reduced the levels of inflammatory markers C-reactive protein, IL-6, ferritin, LDH and D-dimer, and increased the lymphocyte counts	Sanchez-Guijo et al. [64]
MenSCs (allogeneic)	Case report	2 confirmed cases of COVID-19	MenSC transplantation increased the number of CD4^+^ lymphocytes and decreased the expression of inflammatory markers. After transplant treatment, both the SAO_2_ and PO_2_ improved, and chest CTs showed the adsorption of bilateral pulmonary exudates	Tang et al. [66]
Immune-and-matrix- regulatory cells (IMRCs) (Allogeneic)	Phase 1 clinical trial	27 COVID-19 patients who demonstrated pulmonary fibrosis pathology	The pulmonary fibrotic lesions were significantly reduced, and the haematological and clinical chemical parameters remained within the normal range; No tumour markers were detected in the serum	Wu et al. [67]
Cardiosphere-derived cells (CDCs) (allogeneic)	Case series	6 critically ill COVID-19 patients (age range of 19–75 years)	All patients survived with 4 discharged and 1 still on respiratory support compared to 18% mortality in control group. Results were well correlated with diminished levels of ferritin and absolute lymphocyte counts, still suggesting the role of cell-based therapies in modifying the immune responses	Singh et al. [69]
Exosomes derived from BMMSCs (allogeneic)	Prospective nonblinded nonrandomized primary safety trial	24 COVID-19 Patients (18–85 years)	Significant reduction in absolute neutrophil count (p-value < 0.001) with alleviated levels of acute phase reactants, C-reactive protein, downregulating cytokine storm and restoring immunity again implying a key action on immune functions	Sengupta et al. [72]

BMSCs, bone marrow mesenchymal stem cells; hUC-MSCs, human umbilical cord derived MSCs; hWJCs, human umbilical cord Wharton’s jelly derived MSCs; AT-MSCs, adipose tissue derived MSCs; MenSCs, menstrual blood derived MSCs; IMRCs, immune-and-matrix-regulatory cells; CDCs, allogeneic cardiosphere-derived cells

### Bone marrow mesenchymal stem cells (BMSCs)

The first study for stem cell treatment in COVID-19 by Leng et al. reported that 7 COVID-19 patients improved their functional outcomes and promoted rehabilitation after giving intravenous clinical grade MSCs [57]. Seven COVID-19 patients (1 critically severe, 4 severe, 2 common type) were recruited by the Beijing YouAn Hospital in China from January 23, 2020 to February 16, 2020. Each patient received an intravenous infusion of 1 × 10^6^ MSCs per kilogram of body weight. No acute infusion-related adverse or allergic reactions were observed within two hours after transplantation. Before the MSC transplantation, the patients presented with high fever, weakness, shortness of breath and hypoxia. However, at 2–4 days after transplantation, all of the symptoms had disappeared, and the lung function had improved significantly in all patients. In addition, this study suggests that the absence of ACE2 and the high expression of certain trophic factors may be the immunomodulatory mechanism of MSCs.

### Human umbilical cord derived MSCs (hUC-MSCs)

The umbilical cord (especially Wharton’s jelly) is different from the bone marrow and contains a high concentration of MSCs. It is one of the most abundant sources of MSC. MSCs derived from the human umbilical cord (hUC-MSCs) can be extracted noninvasively, and the cells proliferate rapidly, making them the most suitable stem cells for the treatment of COVID-19. Alturi et al. reported a patient treated with hUC-MSCs (3 doses each of 50 million 3 days apart with allogenic umbilical cord MSCs), and the patient was a 65-year-old COVID-19 patient from China [58]. After treatment, there were no known adverse or hypersensitivity reactions. Wang et al. reported a phase I clinical trial of hUC-MSCs in the treatment of COVID-19 [59]. hUC-MSCs were infused intravenously three times on days 0, 3 and 6 in moderate and severe patients (3 × 10^7^ cells per infusion). No serious infusion-related adverse events were observed. A severe patient in the control group still had significant lung lesions upon discharge. In patients treated with hUC-MSCs, the PaO_2_/FiO_2_ ratio improved, and the IL-6 level decreased. Chest CTs showed that the lung lesions of patients with hUC-MSC infusion were well controlled within 6 days and disappeared completely within 2 weeks.

Shu et al. reported the efficacy and safety of the infusion of hUC-MSCs for severe COVID-19, with an intravenous infusion of UC-MSCs performed in 12 patients with severe COVID-19 [60]. The results showed that compared with the control group, the hUC-MSC infusion could reduce the levels of inflammatory CRP and IL-6, accelerate the recovery of the lymphocyte count and shorten the absorption period of lung inflammation. In addition, the hUC-MSC infusion improved the clinical symptoms of weakness and fatigue, tachypnea, and hypoxia. The 28-day mortality of the treatment group was 0, while that of the control group was 10.34%.

Similarly, Peng et al. reported the case of a 66-year-old female patient who received hUC-MSC treatment [61]. The patient developed sore throat, cough, fever and SARS-CoV-2 after contact with a COVID-19 patient. The patient continued to deteriorate with conventional treatment, but her pulmonary function improved significantly after an intravenous infusion of convalescent plasma (CP) and UC-MSCs. A few days later, her SARS-CoV-2 test was negative, and she recovered and was discharged from the hospital. Another study by Liang et al. presented a case of a severe COVID-19 patient aged 65 years treated with hUC-MSCs [62]. The patient received 3 intravenous injections of hUC-MSCs (5 × 10^7^ cells each), and the allograft showed good immune tolerance. After the second intravenous injection, neutrophils had decreased, lymphocytes had increased, and CD4^+^/CD8^+^ T cells returned to normal levels. In addition, the patient’s virology, pulmonary imaging, and clinical biochemical laboratory indicators were significantly improved.

Zhang et al. also reported a case of a 54-year-old man with a history of cough, chest tightness and fever for 4 days [63]. He had no specific medical history other than a 2-year history of diabetes. Due to the deterioration of the patient's condition and the serious organ damage caused by inflammation, human umbilical cord Wharton’s jelly derived MSC (hWJC) adoptive transfer therapy was proposed under the advice and guidance of the expert group. During the hWJC injection, other conventional therapies were used as usual. No acute infusion-related or allergic reactions, delayed hypersensitivity or secondary infection were observed within 2 h after transplantation. Two to seven days after transplantation, the symptoms of discomfort disappeared, the patient's inflammatory state and lung function improved significantly, and he was discharged from the hospital 7 days after treatment.

### MSCs derived from other sources

COVID-19 patients treated with MSCs derived from adipose tissue (AT-MSCs) were reported by Sanchez-Guijo et al. [64]. Thirteen adult patients with COVID-19 under invasive mechanical ventilation who had received antiviral and anti-inflammatory therapy received allogeneic AT-MSC treatment in this study. Based on the patient standard of 0.98 × 10^6^ AT-MSCs/kg, 10 patients received 2 doses, two patients received 1 dose, and another patient received 3 doses. There were no adverse events associated with the stem cell therapy. After treatment with the AT-MSCs, the inflammatory parameters such as C-reactive protein, IL-6, ferritin and LDH were decreased and the lymphocytes were increased. Finally, clinical improvement was observed in 9 patients (70%), of which 7 patients were discharged from the ICU.

MSCs derived from menstrual blood (MenSCs) have typical MSC characteristics such as self-cloning, rapid proliferation, and pluripotency [65]. MenSCs can be easily obtained from discarded menstrual blood in a noninvasive way, and can be obtained periodically and transplanted without trauma or ethical risk. Therefore, MenSCs have a greater clinical application potential than BMSCs and ADSCs. Tang et al. reported that MenSCs can also be used as an alternative treatment for COVID-19, especially in ARDS patients [66]. MenSC transplantation increased the number of CD4^+^ lymphocytes and decreased the expression of inflammatory markers. After transplant treatment, both the SAO_2_ and PO_2_ improved, and chest CTs showed the adsorption of bilateral pulmonary exudates.

Pulmonary fibrosis is a serious complication in patients with COVID-19. Wu et al. reported a population of MSC-like stem cells derived from human embryonic stem cells (hESCs), which they named immune-and-matrix-regulatory cells (IMRCs) [67]. Based on the success of IMRCs in the study of acute lung injury in a mouse model, they conducted a phase 1 clinical trial using hESC-IMRCs in patients with pulmonary fibrosis due to COVID-19 [68]. In this trial, 27 COVID-19 patients with varying degrees of pulmonary fibrosis and respiratory symptoms received intravenous infusions of hESC-IMRCs at a dose of 3 × 10^6^ cells/kg body weight. All patients showed clinical improvement at 84 days after treatment with the hESC-IMRC. The pulmonary fibrotic lesions were significantly reduced, and the hematological and clinical chemical parameters remained within the normal range. None of the patients receiving cell therapy had any associated abnormal reactions or adverse events. In addition, no tumor markers were detected in the serum, indicating that the intravenous infusion of hESC-IMRCs is safe for COVID-19 patients with lung injury.

#### Other stem cells

Allogeneic cardiosphere-derived cells (CDCs) are a type of cardiac stem cells that have a strong ability to differentiate and regenerate the vasculature in vitro. After an injection into animals, CDCs can colonize, migrate and differentiate well. Singh et al. assessed the safety and efficacy of another cell-based therapy (CAP-1002, derived from CDCs) in critically ill patients diagnosed with COVID-19 in 2019 [69]. They evaluated six patients in the age range of 19–75 years who had an intravenous infusion of CAP-1002, and no adverse events related to the administration were observed. The six patients with severe COVID-19 tolerated the CAP-1002 intravenous infusion well, and four of them were discharged. However, the mortality of 34 critically ill patients in the control group was 18%. These results support the safety of CAP-100 in COVID-19 patients.

#### Exosomes Derived from MSCs

Recent studies suggest that the therapeutic effects of MSCs, especially those that are immunomodulatory, can be largely attributed to paracrine effectors [70, 71]. Exosomes (EXOs) released by MSCs are one of the important components of paracrine factors. Thanks to its remote targeting and stability, some EXOs can replace MSCs from which they are derived to perform similar therapeutic functions. In a prospective nonrandomized open-label cohort study conducted by Sengupta et al. the safety and effectiveness of allogeneic bone marrow MSC-derived exosomes (ExoFlo) was explored in the treatment of patients with severe COVID-19 [72]. Twenty-four patients aged 18–85 received a single 15 mL intravenous dose of ExoFlo and were evaluated for safety and efficacy within 14 days after treatment. After the intravenous infusion, there was no adverse reaction attributable to the ExoFlo treatment. The study had a survival rate of 83%, a cure rate of 71%, and a mortality rate of 16% (not related to ExoFlo treatment), while 13% of patients remained seriously ill. After the ExoFlo administration, in addition to the reduction in acute phase reactants, the reduction in neutrophils and lymphocytes was significantly improved.

Compared with MSCs, MSC-EXOs can be isolated from patients in a non-invasive manner. They present the composition, physiological state and characteristics of original MSCs and contain unique bioactive molecules [73]. Importantly, MSC-EXOs are small and easy to circulate, with low immunogenicity, long half-life, high stability and efficiency [74]. Moreover, use of EXOs is a cell-free therapy, which would minimize safety concerns upon injecting live cells by avoiding the transfer of cells that may have mutated or damaged DNA and the risk of malignant transformation associated with MSC infusion [75]. However, MSC-EXO therapy is still in its early stage and many questions should be addressed before it can be widely used in clinic. For example, the qualities of MSC-EXOs need to be controlled, as the characteristics of EXOs depend on the state of MSCs, which decides the therapeutic effect [71]. Only when guidelines and standards for efficacy and safety issues are well established for the therapeutic effect of EXOs, the clinical application of MSC-EXOs could be accelerated.

## Mechanisms of stem cell therapy for COVID-19

Immune abnormalities are the main reason for the progression of severe COVID-19 patients. SARS-CoV-2 rapidly replicates after invading the body, triggering the immune system to release inflammatory cells and antibodies. In most cases, the virus is smoothly cleared by the immune system of the body. However, after SARS-CoV-2 infection, the immune regulatory network of the body is unbalanced, and a large number of inflammatory cytokines are released, such as tumor necrosis factor (TNF)-α, granulocyte colony-stimulating factor (GCSF), inter-leukin (IL)-1α, IL-12, IL-1β, IL-2, IL-6, IL-7, IL-10 (Th2) and IFN-γ (Th1), resulting in cytokine storm syndrome (CSS) [21, 23]. The mechanism of the cytokine storm causes further ARDS, acute cardiac injury and secondary infection, leading to generalized sepsis and multisystem failure. Accumulating studies have shown significant increases in plasma cytokine levels occur in patients with severe COVID-19, suggesting that CSS plays an important role in SARS-CoV-2 deaths. Avoiding the CSS may be the key for the treatment of COVID-19 infected patients.

MSCs possess extensive immunoregulatory abilities and can regulate both the innate immune system and the adaptive immune system, making them the most promising cell-based therapy for COVID-19 [50, 76]. As indicated in Fig. 1, MSCs can secrete many types of soluble factors, such as nitric oxide, indoleamine 2,3-dioxygenase, prostaglandin E2, TGF-β and IL-10 by paracrine secretion, as well as the release of extracellular vesicles and EXOs to suppress excessive immune responses and CSS. In addition, MSCs can also make direct interactions with various immune cells including lymphocytic T cells, B cells, macrophages, neutrophils and NK cells to regulate the intensity and balance of the immune response [77]. Moreover, studies have found that adult cells only produce interferon when the virus invades, which activates hundreds of genes that resist viral infection and recruits immune cells to resist the viral infection, while stem cells can be independent of interferon and can continuously activate many antiviral genes.

In addition to regulating immunity, MSCs also have promising advantages in the treatment of lung injury and repair caused by viral infections. MSCs have the potential function of multipotent differentiation and can produce a variety of cytokines and growth factors, which can treat or repair virus-induced lung tissue damage by affecting the PI3K/Akt, Wnt, NF-κB and other cell signaling pathways [52]. After an intravenous injection, some of the MSCs homing in the lungs can differentiate into alveolar epithelial cells and pulmonary vascular endothelial cells [78], which can increase the secretion of alveolar surface active substances and promote vascular regeneration, thus promoting injury repair. In addition, MSCs can also promote the repair of other damaged tissues in patients with COVID-19. Studies have shown that MSCs activate a variety of repair mechanisms by secreting cytokines, including anti-inflammatory and anti-apoptotic effect factors, and promote angiogenesis to promote the repair of the kidney and intestine.

## Challenges in stem cell therapy of COVID-19

MSC-mediated immunomodulation and regenerative therapy have been suggested for the treatment of COVID-19. However, there are still many challenges in treatment using MSCs. The main technical challenge is the low homing efficiency of the MSCs [79]. Intravenous injection of MSCs shows low homing efficiency, and the cells are trapped in pulmonary capillaries. This process can be partially explained by the insufficient production of homing factors (such as CXCR4) on MSCs [80, 81]. According to reports, the presence of MSCs gradually leads to a significant decrease in the expression of homing factors during in vitro propagation [82]. Several strategies have been used to improve the homing ability of MSCs, including targeted drug delivery, gene modification, magnetic guidance, in vitro priming, cell surface modification, and radiotherapy techniques [83, 84]. In addition, other factors include the source and dose of MSCs, the time window of administration, the route of administration, the frequency of administration, cell isolation and growth strategies, etc., which require further exploration and optimization. MSCs also have a low internal survival rate, and there is donor variability. These issues need to be urgently solved.

MSC-mediated therapy of COVID-19 also faces problems such as immunogenicity, a limited number of cells, and the possible risks of infusion [85]. In terms of safety and efficacy, the clinical use of autologous BMSCs is the best method, but it takes quite a long time to produce clinically relevant numbers of stem cells, which is not always feasible in the current COVID-19 emergency. Rapidly preparing the optimal number of clinical grade MSCs and providing them during treatment is an important challenge for stem cell therapy [86]. For example, BMSCs are scarce in primary tissues, so these cells need to be expanded in vitro to obtain hundreds of millions of cells as a therapeutic dose. Such cell expansion takes several weeks. In the current urgency of the epidemic, managing time, cost, GMP-grade reagents and appropriate quality testing are another challenge [87]. In addition, the genomic stability and regenerative potential of expanded MSCs may be compromised, which raises another concern about the safety of expanded MSCs for clinical use. In addition, stem cell therapy is expensive, and most people may not be able to afford it [88].

MSC transplantation for the treatment of COVID-19 may also have infusion risks: first, there are product risks related to the stem cell quality standards and production processes, and these include fever and allergic reactions caused by the heterologous substances such as serum and the culture medium added during the culturing process. The second is the risk related to the route of administration of stem cells and the distribution in the body after infusion [89, 90]. After intravenous administration of stem cells, they first pass through the pulmonary circulation. An impaired lung function may further increase the burden of the lung microcirculation, resulting in decreased gas exchange and an increase in the heart load. Close monitoring of respiratory and circulatory indicators after infusion can help to detect possible infusion risks in time.

## Stem cells in modeling multiorgan infection by SARS-CoV-2

In addition to being used for therapy, stem cells also play a key role in modeling COVID-19 disease. In the past decade, organoid technology has been one of the most important advances in stem cell research. Three-dimensional (3D) organoids are in vitro tissue models derived from stem cells, including adult stem cells (ASCs) or pluripotent stem cells (PSCs), which contain multiple organ-specific cell types, can truly simulate the physiological structure and function of organs in vivo and are called "organs in-a-dish" [91]. At present, a variety of organoids have been successfully established, such as retinal-, lung-, brain-, and gastrointestinal-organoids [92–96]. They have been rapidly and widely used in many applications, including basic research and translational medicine.

The SARS-CoV-2 virus that caused the COVID-19 pandemic primarily targets the respiratory epithelium and causes acute respiratory distress syndrome. Clinical studies over the past year have shown that SARS-CoV-2 may cause multiple organ dysfunctions in patients, and the presence of the virus has been detected in various organ systems [97]. To investigate the possibility of SARS-CoV-2 infection in multiple organs, stem cell models of different organ systems, including the lung, intestine, heart, and brain, can serve as a tool for the direct study of multiple organ damage in COVID-19 (Table 2).

**Table 2:** Stem cell models for SARS-CoV-2

Stem cell model	Cell types	Culture method	Immune response	Key findings	References
ASCs	Liver bile duct-derived progenitor cells	3D	Not discussed	The liver damage in COVID-19 patients might result from direct cholangiocyte injury and consequent bile acid accumulation induced by viral infection	Zhao et al. [98]
ASCs	Alveolar type 2 cells	3D	Type I response in both studies with an MOI of 1 and ISG stimulation	WNT activity is crucial for hAT2 maintenance; AT2s express a SARS-CoV-2 receptor, ACE2, and are sensitive to virus infection; Low-dose IFN pre-treatment blocks SARS-CoV-2 replication in alveolospheres	Youk et al. [26], Katsura et al. [99] and Tindle et al. [100]
ASCs	Intestinal stem cells	2D	IFNL2 and IFNL3 were highly induced and type I-III IFN response at 24 h after inoculation with an MOI of 3	Established the first expandable organoid culture system of bat intestinal epithelium	Zhou et al. [101]
ASCs	Primary gut endothelial stem cells	Pseudo-stratified layer	Increase in interferon genes 72 h post infection with an MOI of 0.1	Intestinal epithelium supports SARS-CoV-2 replication; SARS-CoV-2 induces a stronger interferon response than SARS-CoV in HIOs	Lamers et al. [103]
ASCs	Hepatocytes	3D	Chemokine, IL-17, TNF and NFκB signaling at MOI 0.1 at 24 h	Human hepatocyte organoids are permissive to SARS-CoV-2 infection	Yang et al. [112]
ASCs	Cholangiocytes	3D	Chemokine and IL-17 signaling pathway activated at MOI 0.1 at 24 h	Human cholangiocyte organoids are permissive to SARS-CoV-2 infection	Yang et al. [112]
hPSC derived	colonoids	3D	TNF and IL-17 signatures reported after 24 h with an MOI of 0.1	Identified FDA-approved drug candidates, including imatinib and mycophenolic acid, as inhibitors of SARS-CoV-2 entry	Han et al. [104]
hPSC derived	alveolar type II-like cells; enterocytes		TNF, IL-17 signaling, and cytokine-cytokine Receptor interaction at MOI 0.01 at 24 h	Identified entry inhibitors of SARS-CoV-2, including imatinib, mycophenolic acid (MPA), and quinacrine dihydrochloride (QNHC)	Chen et al. [105]
hPSC derived	Airway cells	3D	IL-6 and IL-18 significantly up-regulated at MOI 0.01 at 24 h	Synergistic Effects of Anti-inflammatory Macrophages with ACE2 Inhibition Against SARS-CoV-2	Duan et al. [106]
iPSC derived	Alveolar type 2 cells	3D	Delayed type I interferon response with an MOI of 5 and ISG stimulation	SARS-CoV-2 infection of pluripotent Stem Cell-Derived Human Lung AT2 Cells Elicits a Rapid Epithelial-Intrinsic Inflammatory Response	Huang et al. [107]
hPSC derived	Cardiomyocytes	2D	Type I interferon response and ISG stimulation at MOI 0.01	Androgen Signaling Regulates SARS-CoV-2 10 Receptor Levels and Is Associated with Severe COVID-19 Symptoms in Men	Samuel et al. [108]
iPSC derived	Neurons	2D/3D	Not discussed	Neurospheres were permissive to SARS-CoV-2 infection and supported productive virus replication	Zhang et al. [110] Ramani et al. [111]
hPSC derived	Microglia	2D	Not discussed	hPSC-derived microglia cells are permissive to SARS-CoV-2 infection. hPSC-derived cells or organoids show similar chemokine responses as COVID-19 tissues	Yang et al. [112]
hPSC derived	Endocrine	3D	Chemokine induction at 24 h after MOI 0.01	hPSC-derived pancreatic endocrine cells are permissive to SARS-CoV-2 infection	Yang et al. [112]
hPSC derived	Cardiomyocytes	3D	IL-11, IL-1B significantly up-regulated at MOI 0.1 at 72 h	hPSC-derived cardiomyocytes are permissive to SARS-CoV-2 infection	Perez-Bermejo et al. [113], Bojkova et al. [114], Sharma et al. [115] and Yanagida et al. [116]
iPSC derived	Capillary; kidney	3D	Not discussed	SARS-CoV-2 can directly infect engineered human blood vessel organoids and human kidney organoids. hrsACE2 can significantly block early stages of SARS-CoV-2 infections	Monteil et al. [117]

ASCs, adult stem cells; iPSC, induced pluripotent stem cell; MOI, multiplicity of infection; hAT2, human lung alveolar type 2; ISG:IFN-stimulated gene; ACE2, angiotensin converting enzyme 2; hrsACE2, human recombinant soluble ACE2; hSIOs, human small intestinal organoids

### Adult stem cell derived model

The first study of using organoids to model SARS-CoV-2 infection was completed by Zhao et al. [98]. This study explored the mechanism by which SARS-CoV-2 attacks the human liver. They used liver bile duct-derived progenitor cells embedded in Matrigel to assemble long-term extensible 3D structures known as liver ductal organoids. Based on this liver organoid model, it was confirmed that SARS-CoV-2 can infect bile duct cells and downregulate the expression of genes related to cellular tight junctions and bile acid transport in bile duct tissue, suggesting that bile duct dysfunction may be the cause of liver injury in some COVID-19 patients. In addition, the expression of apoptosis-related factors in SARS-CoV-2-infected organoids was upregulated, which provides an important tool for the study of novel coronavirus cell affinity, pathogenic mechanisms and subsequent drug development.

SARS-CoV-2 is thought to be transmitted through the respiratory tract and infect lung. Youk et al. developed a long-term feeder free (3D) culture technique to extract human lung alveolar type 2 (hAT2) cells from primary human lung tissue to study the response to SARS-CoV-2 infection [26]. It was found that the virus replicated rapidly in the infected cells, and the expression of interferon related genes and proinflammatory genes increased. This model provides a useful tool for the study of the pathogenesis of SARS-CoV-2. Similarly, Katsura et al. established a feeder free, scalable, chemically defined and modular alveolar circle culture system for the proliferation and differentiation of human alveolar type 2 cells/pneumocytes extracted from primary lung tissue [99]. The cultured lung cells expressed ACE2 and therefore could be infected by SARS-CoV-2. Alveolar spheres infected with the virus can reflect the characteristics of the COVID-19 lungs, including interferon (IFN)-mediated inflammatory reactions, loss of surfactant proteins, and apoptosis. The results of the study by Tindle et al. also validated a human lung model of COVID-19, which can be immediately used to study the pathogenesis of COVID-19 and to review new therapies and vaccines [100].

Additionally, Zhou et al. found that SARS-CoV-2 can infect the intestinal organs of humans and bats and can maintain strong viral replication ability [101]. In this study, bat and human intestinal organoids were constructed by intestinal stem cells using a previously reported method [102]. Then, nasopharyngeal samples obtained from COVID-19 patients were cocultured with human or bat intestinal organoids, and the cultured supernatant was used to reinfect the organoids. The test results showed that the viral load in the intestinal organs increased rapidly over time. In addition, the researchers found that both the ACE2 and TMPRSS2 expression levels required for SARS-CoV-2 to invade the host cells were significantly improved in differentiated human intestinal organoids and the distribution of ACE2 and TMPRSS2 protein in bat intestinal organoids. Similarly, Lamers et al. established a 3D model of human small intelligent organisms (hSIOs) from primary gut endothelial stem cells and carried out viral infection experiments [103]. These research results showed that in hSIOs, intestinal cells are easily infected by SARS-CoV and SARS-CoV-2 and support SARS-CoV-2 replication.

### PSC derived stem cell model

Han et al. first reported the use of hPSC-derived lung organoid models to study COVID-19 disease and the use of this model in the screening of therapeutic drugs [104]. Three drugs approved by the FDA for COVID-19 infection were successfully verified as effective in this pulmonary organ model. Before this, SARS-CoV-2-infected cell lines were used to screen drugs. However, it is difficult for the cell lines to simulate the behavior of tissue cells with regard to the pathophysiological process after viral infection, and organoids induced by hPSCs can partially simulate the real organ signals, which makes the research of the screened drugs have more clinical significance. For this, Han et al. also developed hPSC-derived lung and colonic organoids (hPSC-LOs and hPSC-COs) to explore the response of colonic cells to SARS-CoV-2 infection and optimized as in vitro platforms for high throughput drug screening. Using these platforms, they identified entry inhibitors of SARS-CoV-2, including imatinib, mycophenolic acid (MPA) and quinacrine dihydrochloride (QNHC), which significantly inhibit SARS-COV-2 infection of both hPSC-LOs and hPSC-COs on physiologically relevant levels [105].

Duan et al. established a coculture system of lung and macrophages by directional differentiation of hSCs and simulated the host pathogen interaction and immune response caused by SARS-CoV-2 infection [106]. This study found that in the early stages of infection, M2 macrophages can eliminate SARS-CoV-2 by preventing viruses from entering target cells and enhancing the anti-inflammatory effects of macrophages. They also praised the hPSC induction model for providing a large number of cells with a genetic background, thus avoiding concerns about histocompatibility and facilitating reliable mechanical and therapeutic research.

Additionally, Huang et al. simulated the initial infection of alveolar epithelial cells by SARS-CoV-2 using AT2s derived from human induced pluripotent stem cells (hiPSCs) that had been adapted to an air–liquid interface culture [107]. They found a type of rapid transcriptional change in the infected cells, characterized by a shift to an inflammatory phenotype, including the upregulation of NF-κB signal transduction and the disappearance of mature alveolar programs. Drug testing confirmed the effectiveness of remdesivir and TMPRSS2 protease inhibition and verified the hypothetical mechanism of virus entry into alveolar cells. The stem cell model system of this research group revealed the intrinsic cellular response of lung target cells to SARS-CoV-2 infection, which is helpful for drug development.

Samuel et al. highlighted the potential of organoids derived from hESCs for the in-depth study of diseases with specific physiological and pathological characteristics and provided an excellent model for the development of new drugs and precision therapy [108]. Their results showed that androgen signaling is a key regulator of ACE2 levels, and treatment with anti-androgen drugs reduced the expression of ACE2 and protected the lung organs derived from hESCs against SARS-CoV-2 infection. These findings provide insights into the mechanism of disproportionate male susceptibility to the disease and identify anti-androgen drugs as candidate therapies for COVID-19. Pei et al. also generated human lung airway and alveolar organoids from hESCs. These organoid systems can not only simulate SARS-COV-2 lung infection as a pathophysiological model to study SARS-COV-2 infection, but also be used for discovery and testing of targeted drugs for COVID-19 treatment [109].

The first evidence of direct infection of SARS-CoV-2 in human brain organoids came from the team of the University of Hong Kong, who used pluripotent induced stem cells to create a human brain organoid. The extensive expression of viral proteins and infectious virus particles was detected in the neurospheres and brain organoids infected by SARS-CoV-2. SARS-CoV-2 infection was localized in TuJ1 (neuron marker) and Nestin (neural stem cell marker) positive cells in the 3D human brain organoids, indicating that SARS-CoV-2 can directly affect cortical neurons and neural stem cells [110].

Then, Ramani et al. used iPSC-derived human brain organoids to establish a test system for SARS-CoV-2 infection, which provides an indication of the potential neurotoxic effects of SARS-CoV-2 [111]. Through the development of a broad platform using a system-wide human cell lineage and organoids, Yang et al. found that pancreatic α and β cells, liver organs, cardiomyocytes and dopaminergic neurons could all be infected with SARS-CoV-2 by pseudoentry and live SARS-CoV-2 system [112].

Some researchers have also used human induced pluripotent stem cell-derived cardiomyocytes (hiPSC CMS) as a model to study the mechanism of SARS-CoV-2 cardiomyocyte-specific infection. SARS-CoV-2 infection was highly tolerated in liver organoids, and SARS-CoV-2 infected cardiomyocytes in an ACE2- and cathepsin-dependent manner in vitro, which could be inhibited by the antiviral drug remdesivir [113–116].

In addition, Monteil et al. established human vascular and renal organoids that were induced by iPSCs in vitro and found that organoid infection caused by SARS-CoV-2 can be inhibited by human recombinant soluble ACE2 (hrsACE2) and that hrsACE2 inhibits SARS-CoV-2 infection in a dose-dependent manner [117]. It has been directly demonstrated that hrsACE2 can be used as an antiviral drug for the treatment of COVID-19.

## Conclusion and future perspectives

In general, vaccination is undoubtedly the best choice to fight against the COVID-19 pandemic. If universal vaccination can be achieved, an immune barrier will be established in population to effectively block the continuous transmission of SARS-COV-2. However, data from current clinical studies cannot determine the duration of COVID-19 vaccine protection, and no vaccine can achieve 100% protection. A small number of people even achieve no protective efficacy from vaccination, or are still infected, which is related to both the characteristics of the vaccine itself and the individual health condition of the recipient. Therefore, other therapies based on scientific knowledge are also viable options in the future, such as stem cell therapy. Previously, stem cell therapy was attempted in patients with severe H7N9 avian influenza, and good results were achieved. Even if drugs kill and repel the virus, they may damage the lungs, causing sequelae. However, stem cell therapy can repair the lungs if it is effective and can transplant a new lung, thus fully restoring the respiratory function. Recently, a large number of preliminary studies have shown that stem cells show safety and effectiveness in the treatment of severe COVID-19 disease, showing good potential.

In addition to a large number of in vivo and external studies, thus far, more than 100 clinical trials for COVID-19 are on the international clinical research registration website (clinicaltrials.gov), and most of the application projects are focused on the target population for critical COVID-19 patients. Stem cells were derived from tissues such as the umbilical cord, umbilical cord blood and dental pulp, with umbilical cord MSCs being the most commonly used. hUC-MSCs are abundant and convenient to prepare, are considered to have the functions of inhibiting the inflammatory response, improve damaged tissue nesting and repair, and play an important role in the treatment of autoimmune diseases. As mentioned earlier in this study, allogeneic MSCs have been basically used to treat COVID-19 patients, and no serious adverse events associated with MSC administration were observed. On the one hand, after MSCs infusion, MSCs are able to increase the number of lymphocyte and regulatory DCs to improve their antiviral capacity which down-regulates levels of the dominating markers of inflammation and ROS to diminish the inflammation and oxidative stress such as the C-reactive protein and pro-inflammatory cytokines including IL-6, TNFα, IL-8. Meanwhile, the number of infiltrated immune cells decreases significantly [62]. On the other hand, MSCs can increase the level of IL-10 which functions as an anti-inflammatory protein to activate regulatory cells such as Tregs [118], and reduce the expression levels of TNF-α as well as IL-12 in the blood. In addition, there are a number of projects using the combination of immune cells or inhibitory drugs with stem cell therapy interventions, and there are new intervention methods using stem cell-derived EXOs for atomization inhalation therapy.

Traditional virus research tools are cell lines or laboratory animals, but they cannot effectively simulate the infection process of COVID-19 and the real mechanisms within the body. The organoids induced by stem cells can better reflect the effect of SARS-CoV-2 on human tissues because they resemble real tissues, contain multiple cell types after growth, and have a short culture cycle in vitro. The above studies indicate that organoids have become a powerful tool for the study of novel coronaviruses. In recent years, organoid technology has developed rapidly and has been widely used in the study of organ development, the construction of disease models, drug screening and individualized medical treatment.

However, the treatment of COVID-19 with stem cells still faces many difficulties and challenges. Since stem cell therapy is still in the experimental stage, few stem cell therapies have been approved thus far, and a small number of them are experimental treatments used in sympathetic usage. At the same time, the treatment of COVID-19 with stem cell therapy still needs to overcome many technical difficulties. First, the current infection rate of COVID-19 has increased, but the number of patients with no disease and mild disease is increasing, resulting in fewer cases of severe clinical patients. Therefore, it is more difficult to recruit patients for clinical trials. Second, stem cell therapy still lacks preclinical experimental data, especially data from animal models of lung injury caused by SARS-CoV-2 infection. Third, the biological activity, cell dryness and purity of stem cells may change in different production batches, which will affect the final efficacy. Fourth, the therapeutic effect of stem cells from different tissue sources may vary greatly. In addition, the in vitro culture conditions of stem cells and organoids are also important quality control conditions that affect clinical trials.

Overall, although the clinical research of stem cells is still in its infancy, with the continuous exploration of stem cell clinical research and the continuous mining of data, stem cell therapy has broad clinical application prospects and a far-reaching significance. We also hope that the continuous improvement of stem cell therapy can save more COVID-19 patients with severe disease and save more lives.

## Data Availability

Not applicable.

## Abbreviations

COVID-19: Coronavirus disease 2019
SARS-CoV-2: Severe acute respiratory coronavirus 2
ARDS: Acute respiratory distress syndrome
WHO: World Health Organization
MERS: Middle East respiratory syndrome
SARS: Severe acute respiratory syndrome
MSCs: Mesenchymal stem cells
ALI: Acute lung injury
S protein: Spike glycoprotein
hESCs: Human embryonic stem cells
EXOs: Exosomes
ExoFlo: Allogeneic bone marrow mesenchymal stem cell-derived exosomes
CSS: Cytokine storm syndrome
3D: Three-dimensional
PSCs: Pluripotent stem cells
hAT2: Human lung alveolar type 2
hPSC: Human pluripotent stem cell
hSCs: Human stem cells
hiPSC: Human induced pluripotent stem cell
hPSC-LOs: Human pluripotent stem cell-derived lung organoids
hPSC-COs: Human pluripotent stem cell-derived colonic organoids
